# Seasonal Changes of Growth, Immune Parameters and Liver Function in Wild Chinese Sturgeons Under Indoor Conditions: Implication for Artificial Rearing

**DOI:** 10.3389/fphys.2022.894729

**Published:** 2022-04-19

**Authors:** Yueping Zheng, Yong Zhang, Zhe Xie, Paul K. S. Shin, Jianan Xu, Houyong Fan, Ping Zhuang, Menghong Hu, Youji Wang

**Affiliations:** ^1^ International Research Center for Marine Biosciences & College of Fisheries and Life Science at Shanghai Ocean University, Ministry of Science and Technology, Shanghai, China; ^2^ Key Laboratory of Exploration and Utilization of Aquatic Genetic Resources, Ministry of Education, Shanghai Ocean University, Shanghai, China; ^3^ Shanghai Aquatic Wildlife Research Center, Shanghai, China; ^4^ Department of Biology and Chemistry, City University of Hong Kong, Hong Kong, Hong Kong SAR, China; ^5^ Key Laboratory of East China Sea & Oceanic Fishery Resources Exploitation and Utilization, Scientific Observing and Experimental Station of Fisheries Resources and Environment of East China Sea and Yangtze Estuary, East China Sea Fisheries Research Institute, Chinese Academy of Fishery Sciences, Shanghai, China

**Keywords:** Chinese sturgeon, indoor culture, innate immunity, liver function, seasonality, sex differences

## Abstract

Seasonality has a significant effect on the physiology of fish, especially the effect of water temperature changes. In the present study, the growth, innate immune parameters and liver function indices of two rescued wild adult Chinese sturgeons under captive conditions were monitored for 1 year. The results showed that the total annual weight loss rate of the male was −4.58% and the total weight gain rate of the female was 24.12%, in which the weight of both individuals registered highly significant differences in summer, fall and winter (*p* < 0.01). The male Chinese sturgeon also exhibited negative specific growth rates (−0.1 to −0.8%) during spring to fall, whereas positive specific growth rates, ranging from 0.03 to 0.11%, were recorded in the female. Seasonality also affected the innate immune parameters of the two Chinese sturgeons, in which leukocytes had been increasing since spring and C-reactive protein (CRP) content was significantly higher (*p* < 0.05) in summer than fall in both individuals. The CRP level of the male Chinese sturgeon showed a significant increase from fall to winter (*p* < 0.05), suggesting that it may have contracted infection or inflammation during this study period. With the analysis of serum alanine aminotransferase (ALT), aspartate aminotransferase (AST), transaminase (AST:ALT) ratio, alkaline phosphatase, albumin to globulin ratio and triglycerides, it was found that the liver function of the captive Chinese sturgeons was adversely affected along seasonal changes, with the highest degree of liver impairment in winter. In combining observations from growth performance and changes in innate immune and liver function parameters, the present findings deduced that the male Chinese sturgeon under study was more susceptible to seasonal changes than the female. For better indoor culture of adult Chinese sturgeons, monitoring of hematological parameters to detect early signs of inflammation and liver function abnormality should be conducted with routine veterinary care during prolonged captivity.

## Introduction

One of the most important factors affecting seasonal changes in fish behavior is water temperature. In fish studies, temperature change has been a major focus of interest for researchers, including growth and adaptation in immune response. Temperature change can affect the physiology of organisms, leading to long-term impacts on reproduction, growth, and geographic distribution (Qiang et al., 2021). Most marine fish species express life-history changes across temperature gradients, such as faster growth, earlier maturation and higher mortality at higher temperatures (Wang et al., 2020). For example, warming low-land habitats and increasing water temperatures can increase metabolic rates and stress of fish, forcing the rainbow trout *Oncorhynchus mykiss* to migrate upstream to cooler waters and confining their habitat range (Morales-Marin et al., 2019). The increase in temperature also indirectly affects age and size at smolting through its effect on the growth of Atlantic salmon *Salmo salar* and brown trout *Salmo trutta*, in which age at first maturity, longevity and fecundity decreased with increasing temperature, while the size of eggs increased with temperature rise (Jonsson and Jonsson, 2009).

The response of fish to temperature and other environmental changes can be assessed by hematological parameters, such as blood cell number and maturity (Qiang et al., 2021; Seibel et al., 2021). Blood and its components can reflect a variety of diseases, and abnormalities in red blood cells, white blood cells, platelets and coagulation factors are considered primary blood disorders. Such abnormalities in blood cell function or structure may result in anemia, leukopenia, neutropenia, thrombocytopenia, and other blood cell anomalies (Clauss et al., 2008). Blood parameters have been used as indicators of health status in many fish to detect physiological changes caused by stress conditions such as transport, handling, hypoxia, and acclimation (Faggio et al., 2014). Houston *et al.* (1996) found that hemoglobin, hematocrit and erythrocytic volume were slightly lower in rainbow trout *O*. *mykiss* during the warm than cold season as the blood circulation started to accelerate with the increase of temperature, resulting in higher metabolism and increase in oxygen carrying capacity and oxygen demand. Although change in hemoglobin isomorph profiles was apparent in rainbow trout in warm and cold seasons, there is little evidence that such changes are of critical adaptive importance (Houston et al., 1996). However, Burgos-Aceves *et al.* (2019) revealed that leukocyte counts in carp and catfish increased significantly during warm but decreased in cold periods, suggesting that temperature may affect fish hematology and alter hematocyte kinetics. A significant increase in the number of neutrophils in Atlantic salmon *S. salar* has also been reported at high temperatures (18°C) (Pettersen et al., 2005), and a decrease in water temperature from 23 to 11°C over a 24-h period resulted in anemia, although the number of neutrophils in the blood of the channel catfish *Ictalurus punctatus* was not affected (Martins et al., 2011).

The variation of water temperature in indoor culture is consistent with the variation of water temperature in natural, open waters, and the effect of water temperature variation on fish may be species specific and/or due to sex differences. Chinese sturgeon *Acipenser sinensis* is a large anadromous spawning migratory fish, mainly distributed in the coastal shelf waters of southeastern China and the middle and lower Yangtze River. It is one of the species with the lowest latitudes of distribution among 17 species of sturgeons, and adapts to higher water temperatures (Wei, 2019). With its large size, long life span and wide migratory range, Chinese sturgeon is a flagship species in the Yangtze River aquatic ecosystem (Wei, 2020). However, owing to historical overfishing and loss of spawning grounds caused by dam construction, the population of Chinese sturgeons has been significantly in decline and the species is now listed as Critically Endangered on the IUCN Red List (Wei, 2010). Efforts are ongoing to enhance the existing Chinese sturgeon population through studies of their biology and ecology with aims to establish artificially rearing and release of cultured juveniles back into the wild (Wan and Fang, 2003; Wang et al., 2011; Chang et al., 2021). However, studies on the effects of seasonal changes on Chinese sturgeons are still relatively scarce, with only a few reports on the reduction or cessation of feeding by Chinese sturgeons at high temperatures, whereas no investigations on the effects of seasonal changes on innate immune parameters and liver function of Chinese sturgeons have been documented. In the present study, seasonal effect on Chinese sturgeons was investigated by analyzing the changes in growth, innate immune parameters and liver function of both male and female Chinese sturgeons for 1 year under artificial culture conditions. The results of this study would provide further scientific data to support future improvement of indoor rearing conditions for Chinese sturgeons.

## Materials and Methods

### The Study Setup

The present study involved adult wild Chinese sturgeons caught by fishermen: one female (Identification No. 306) on 6 March, 2010 and one male (Identification No. 421) on 21 April, 2011, all gonad development was examined at stage 2. After rescue, they were kept in the Shanghai Aquatic Wildlife Conservation Research Center, Chongming, China. From February 2014 to January 2015, an indoor concrete tank, 18 m long and 15 m wide, with a water depth of 1.6–2.0 m was set up for the study. Filtered groundwater was continuously flowed into and out of the tank in a counterclockwise direction, with oxygen supply from an *in-situ* oxygenator (NR-A212, Ranrong, China) at the bottom of the tank. The setting of the indoor tank was referred to previous studies (Zhang et al., 2007; Zhang et al., 2017), basically meeting the needs of the Chinese sturgeon activities. The study was conducted under natural temperature change and photoperiod conditions without artificial intervention, and water quality parameters of the indoor tank were monitored at 6:00, 12:00, and 18:00 daily. Water temperature and dissolved oxygen were measured with a multi-parameter portable meter (WTW Multi 3630 IDS, Xylem Analytics, Germany), whereas ammonia nitrogen was determined with the salicylic acid spectrophotometric method and nitrite with the naphthylenediamine spectrophotometric method according to the “GB 11607–1989 Fishery Water Quality Standards” in China.

At the start of the study, both Chinese sturgeons were examined and their basic biological characteristics are shown in Table 1. During the study, an area of 5 m × 3 m (length × width) was selected as a fixed feeding area on one side of the tank and the two Chinese sturgeons were fed at regular intervals, three times a day at 7:00, 13:00 and 19:00. Each of the two Chinese sturgeons was first fed with the compound feed containing not less than 40% crude protein produced by Ningbo Tianbang Co., Ltd. (Zhejiang, China), and the remaining compound feed were collected after 30 min. Then 150 g of the fresh bait, composed of the flesh of crucian carp *Carassius carassius* after sterilization in 3% brine, were supplied, and the remaining fresh bait was collected after 30 min. However, over the study period, both Chinese sturgeons did not consume the compound feed. Hence, the feeding amount was only obtained from the difference between the total fresh bait amount offered and the residual bait amount left after the feeding duration, and the ratio between the feeding amount and the total fresh bait amount was used as the daily feeding rate. The growth of the two Chinese sturgeons was also checked monthly, including body weight, full length, body length, and maximum body circumference, and the monthly specific growth rate (SGR) and condition factor (CF) were computed using the following formulae:

$$\mathrm{SGR}\left(\%\right)=100\times \left(\ln\;W_{\mathrm{final}}-\ln\;W_{\mathrm{initial}}\right)/t$$

$$\mathrm{CF}=\left(W/L^{3}\right)\times 100$$

where, W is body weight (g), L is body length (cm), and t is study duration (days).

**TABLE 1:** Basic biological characteristics of the two Chinese sturgeons under study.

Basic biological characteristics	No. 306	No. 421
Sex	Female♀	Male♂
Total length (cm)	242	255
Body length (cm)	201	210
Body weight (kg)	128.5	120
Back bone plate	13	13
Dorsal fin bone plate	1	2
Left lateral bone plate	30	35
Right lateral bone plate	33	36
Left abdominal bone plate	11	10
Right abdominal bone plate	10	12
Preanalfin bone plate	2	1
Postanalfin bone plate	2	2

The swimming speed and direction of the two Chinese sturgeons were also monitored visually over the study period once every 2 h for 12 times daily at a fixed observation point set at one side of the tank, according to the method adapted from Zhang *et al.* (2017). Each observation lasted for about 5 min, in which the swimming speed and direction (either along or against water flow in the tank) of each individual were recorded. For reporting the swimming speed, two points were marked on one side of the tank and the distance was measured, then the time for each individual swam in straight line between these two points was recorded with a timer. The swimming speed was calculated as m/s, and the total observations were averaged to obtain the daily swimming speed. In addition, the counter-flow swimming ratio was computed, based on the frequency in which the individual swam against water flow divided by the total 12 observations registered per day.

### Blood Sample Collection and Pretreatment

Blood was collected at the end of each month during the study, and the collection time was at 14:00. A total of 12 blood collections were completed for each fish. Chinese sturgeon was caught by a stretcher in the tank, the abdomen was turned up, the eyes of the Chinese sturgeon were covered with a stretcher cloth. After its physical condition was slightly stabilized, blood was collected from the caudal artery behind the anal fin using a 10 ml disposable sterile syringe. The collected blood was immediately dispensed into 0.5 ml of EDTA anticoagulated centrifuge tubes (pre-dried) and stored away from light for blood cell sorting. The rest of the blood samples were divided into 2 ml centrifuge tubes and left for 3–4 h at room temperature, protected from light, and then centrifuged for 10 min at 4000 r/min at 4°C using a benchtop microfuge (Microfuge 22R, Backman, United States) after coagulation. After centrifugation, the supernatant was transferred to a clean tube with a pipette and stored in an ultra-low temperature refrigerator at −80°C.

### Erythrocyte and Leukocyte Counts

Total counts for erythrocytes and leukocytes as well as differential leukocytes in the blood samples collected from the two Chinese sturgeons were performed with reagents produced by Nanjing Jiancheng Institute of Biological Engineering (Nanjing, China). For erythrocyte counts, 2 ml of red blood cell dilution was added to a 5 ml centrifuge tube, then 10 μl of EDTA anticoagulated blood was pipetted into the centrifuge tube, mixed well, and sampled on a blood cell counting plate. For leukocyte counts, another pipette was used to add 0.38 ml of leukocyte diluent to 1.5 ml centrifuge tube, followed by pipetting 20 μl of EDTA anticoagulated blood into the leukocyte diluent centrifuge tube, mixed well, and the sample was taken on a hemocytometer plate. For differential leukocyte counts, blood smears from sub-divided blood samples were made immediately with EDTA anticoagulated blood, dried away from light, and stained with Richter-Kimsa composite staining solution. All cell counts were conducted under a light microscope at 40X twice for each fish, and the average value was taken. Differential leukocytes, including granulocytes (neutrophils, eosinophil and basophils), monocytes and lymphocytes, were recorded in percentage.

### Routine Serum Biochemical Parameters

Serum biochemical parameters were measured using a Rittal Selectra_E fully automated biochemical analyzer (Vital Scientific, Netherlands), including C-reactive protein (CRP, immunoturbidimetric method) to indicate response to body inflammation, and other liver function parameters such as serum alanine aminotransferase (ALT, IFCC International Federation of Clinical Chemistry rate method), aspartate aminotransferase (AST, IFCC rate method), alkaline phosphatase (ALP, AMP 2-Amino-2-Methyl-1-Propanol buffer method), albumin (ALB, bromocresol green method), globulin (GLO, immunoturbidimetric method) and triglycerides (TG, GPO-PAP glycerol phosphate oxidase-p-aminophenazone method). All these tests were performed using DENUO series biochemical assay kits produced by Shanghai Deocheng Biotechnology Co. (Shanghai, China) at a detection temperature of 37°C. Chemical reagents for the calibration standards and quality control were purchased from Randox Laboratories-US, Ltd., West Virginia, United States.

### Data Treatment and Analysis

Data for the daily feeding rate, swimming speed and counter-flow swimming ratio obtained for each month were averaged, and together with other monthly data, were categorized into spring (February to April), summer (May to July), fall (August to October) and winter (November to January) seasons and expressed as mean ± SEM. Prior to statistical analyses, data were checked for normality and equality of variance using the Kolmogorov-Smirnov and Levene’s tests. Repeated measures ANOVA was used to analyze the significance of differences in those measured parameters among different seasons of the same individual, followed by the post-hoc Duncan’s multiple range test. Student t-test was employed to assess the significance of differences in such parameters between the male and female Chinese sturgeons in the same season. Results of significant and highly significant differences in these analyses were presented by *p < 0.05 and **p < 0.01, respectively. Statistical analyses were performed using SPSS 24.0 (SPSS Inc., Chicago, United States) and data were plotted by GraphPad Prism 8.0 (GraphPad Software, California, United States) respectively.

## Results

### Changes in Water Quality and Growth-Related Measurements

Throughout the study period, monthly mean water temperature ranged from 13.6 to 26.5°C, dissolved oxygen from 7.42 to 8.93 mg/L, ammonia nitrogen from 0.09 to 0.27 mg/L, and nitrite from 0.01 to 0.08 mg/L. In particular, the seasonal mean water temperature of the indoor tank increased slowly from spring (15.6°C) to reach a peak in summer (26.5°C), and then continued to decrease in fall (24.2°C) and winter (15.4°C). Highly significant differences (*p* < 0.01) in water temperature were noted in different seasons.

**FIGURE 1:**
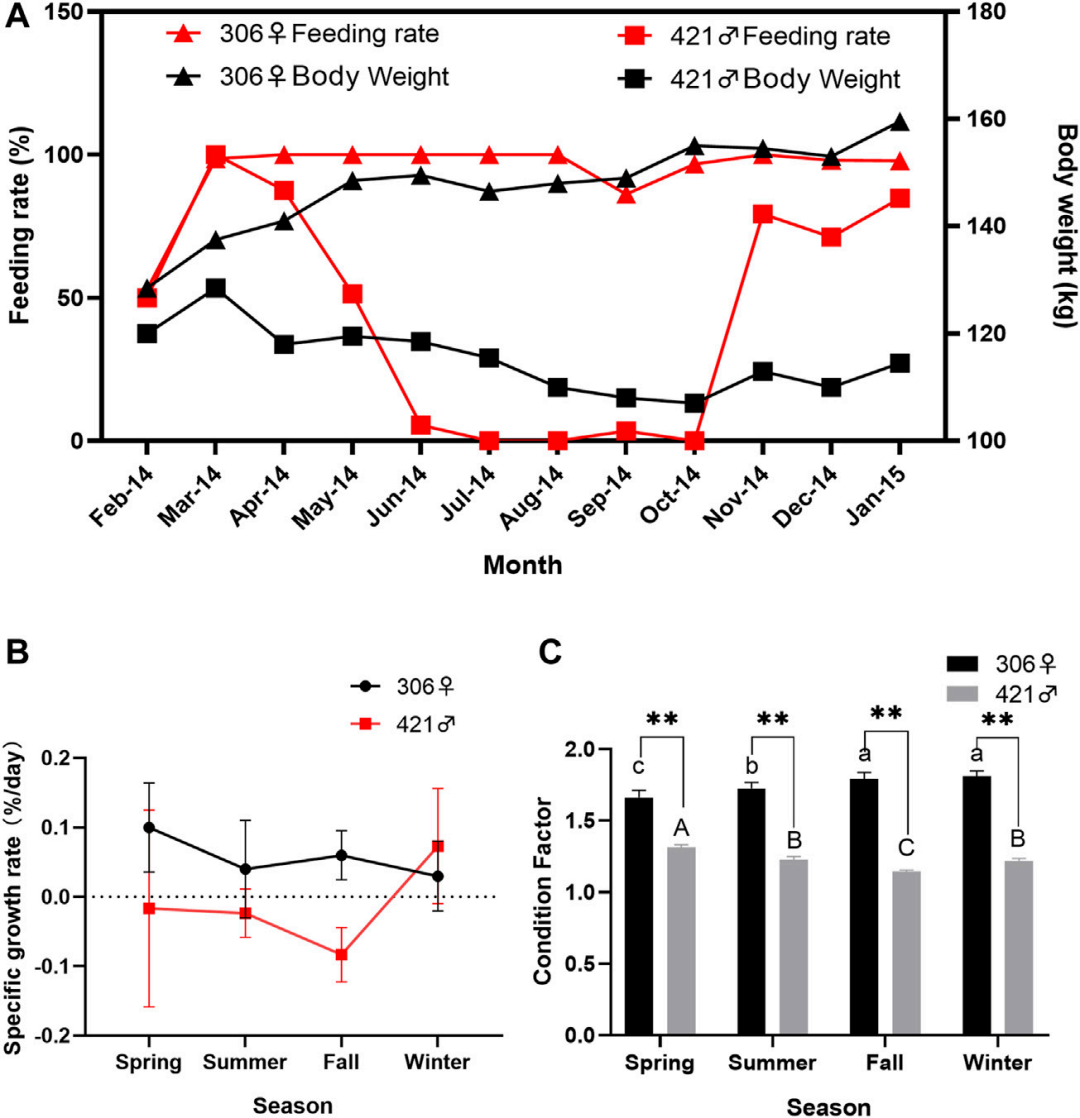
Monthly changes of **(A)** body weight and feeding rate, and seasonal variation of **(B)** specific growth rate and **(C)** Condition Factor of two Chinese sturgeons over the study period. Seasonal data are mean ± SEM (*n* = 3), the seasonal data of sample size (*n* = 3) represents the three monthly data within one season. Highly significant difference (*p* < 0.01) in the parameter between the male and female individuals in the same season is denoted by two asterisks. Different uppercase letters indicate statistically significant differences between the male Chinese sturgeon in different seasons (*p* < 0.05), whereas different lowercase letters indicate statistically significant differences of the female Chinese sturgeon in different seasons (*p* < 0.05). Dashed line represents zero value.

The body weight of two Chinese sturgeons showed an opposite trend. The weight of the female continued to increase, with a total weight gain of 31.0 kg and a total weight gain rate of 24.12% over the study, whereas the weight of the male showed a decrease, with a total weight loss of 5.5 kg and a total weight loss rate of −4.58% over the same period of time (Figure 1A). The weight of both individuals showed highly significant differences in summer, fall and winter (*p* < 0.01). However, the weight of both individuals showed no significant difference in spring (*p* > 0.05). Such differences in body weight change between the two Chinese sturgeons were also reflected in their feeding rate, in which the female had a relatively stable monthly 100% feeding rate except at the start of the study (February 2014), whereas the male showed a continual decrease in its feeding rate from spring to summer and largely ceased to feed in fall until the onset of winter (Figure 1A).

For growth-related measurements, there were minimal changes for both Chinese sturgeons over the study. The full length of the female showed a slight increase from 242 cm at the start to 254 cm at the end of the study, body length from 201 to 208 cm, and maximum body circumference from 122 to 129 cm, respectively. However, the full length of the male exhibited a slight decrease from 255 cm at the start to 251 cm at the end of the study, body length from 210 to 209 cm, and maximum body circumference from 106 to 105 cm, respectively. In terms of specific growth rate, the female Chinese sturgeon began with a higher value at 0.11% in spring but decreased during summer to winter, with values between 0.03 and 0.06%, whereas the male showed consistently negative specific growth rates (−0.1 to −0.8%) during spring to fall except for a slight increase to 0.07% in the winter period (Figure 1B). In comparison of condition factors, the female Chinese sturgeon also had a range between 1.66 and 1.82, significantly higher (*p* < 0.01) than the seasonal values of 1.14–1.31 in the male over the study period and showing an increasing trend from spring to winter whereas the male condition factor decreasing from spring to fall until a slight increase during winter times (Figure 1C).

**FIGURE 2:**
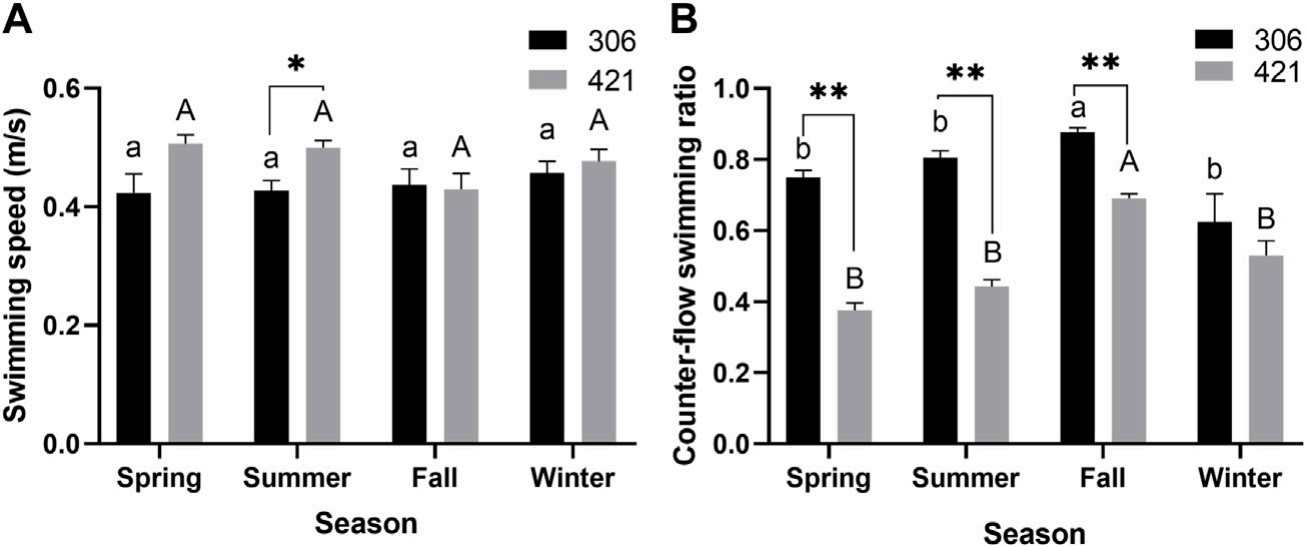
Seasonal changes of **(A)** swimming speed and **(B)** counter-flow swimming ratio of two Chinese sturgeons during the study period. Seasonal data are mean ± SEM (*n* = 3), the seasonal data of sample size (*n* = 3) represents the three monthly data within one season. Significant difference (*p* < 0.05) and highly significant difference (*p* < 0.01) between the male and female individuals in the same season are denoted by one and two asterisks, respectively. Different uppercase letters indicate statistically significant differences among different seasons in the male Chinese sturgeon (*p* < 0.05), whereas different lowercase letters indicate statistically significant differences among different seasons in the female Chinese sturgeon (*p* < 0.05).

The annual mean swimming speed of the male Chinese sturgeon was 0.47 ± 0.12 m/s, with a range of 0.08–1.04 m/s, whereas for the female, this was 0.43 ± 0.10 m/s, with a range of 0.13–1.33 cm/s. The swimming speed of the male was only significantly faster than the female in summer (*p* < 0.05, Figure 2A). However, during the study, the counter-flow swimming ratio of the female recorded (0.71–0.90) was significantly higher than that of the male (0.33–0.71) in spring, summer and fall, and the same swimming ratio of both individuals (male 0.69; female 0.88) were significantly higher in fall than in other seasons (*p* < 0.01, Figure 2B).

### Changes in the Number of Erythrocytes and Leukocytes

**FIGURE 3:**
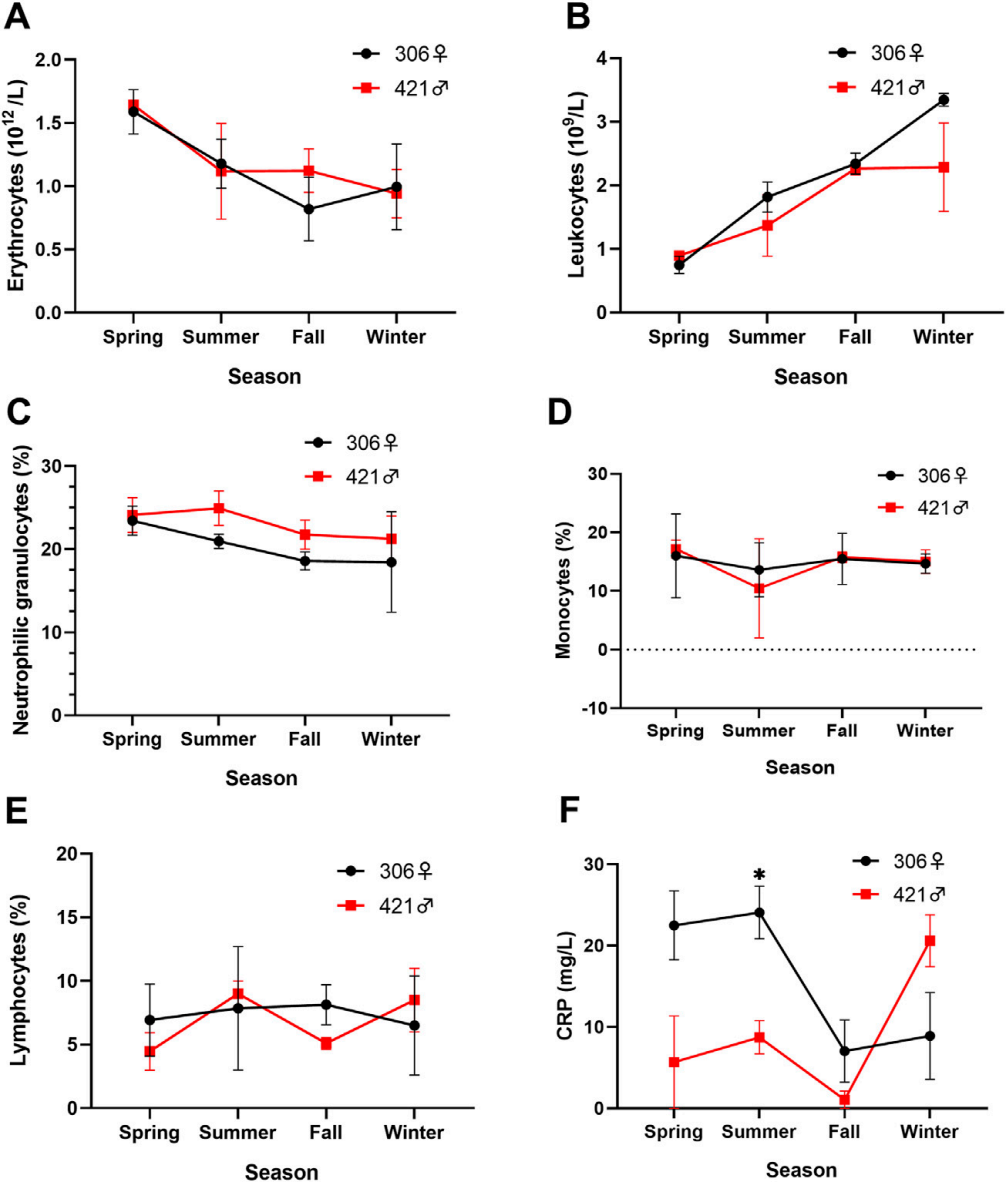
Seasonal variation of **(A)** erythrocytes, **(B)** leukocytes, **(C)** neutrophilic granulocytes, **(D)** monocytes, **(E)** lymphocytes, and **(F)** C-reactive protein (CRP) in the blood of two Chinese sturgeons. Data are mean ± SEM (*n* = 3), the seasonal data of sample size (*n* = 3) represents the three monthly data within one season. Significant difference (*p* < 0.05) in the parameter between the male and female individuals in the same season is denoted by an asterisk. Dashed line represents zero value.

The number of erythrocytes had decreased since the beginning of the study for both Chinese sturgeons, with the highest value in spring (male 1.64×10^12^/L, female 1.59×10^12^/L), the lowest value in winter for the male (0.79×10^12^/L), and the lowest value in fall for the female (0.82×10^12^/L) (Figure 3A). However, the number of leukocytes showed an opposite trend to that of erythrocytes, rising from the beginning of spring (male 0.90×10^9^/L, female 0.75×10^9^/L) and reaching a peak in winter for both individuals (male 2.92×10^9^/L, female 3.35×10^9^/L) (Figure 3B). There was also no sex difference in erythrocyte and leukocyte content for both individuals during the study.

### Changes in Differential Leukocytes and Level of C-Reactive Protein

For the three types of granulocytes in differential leukocyte counts, basophils and eosinophils were largely not detected in the blood smear samples, and the content of neutrophils showed a slight decreasing trend, from 24.1 to 19.2% and 23.4 to 19.0% in the male and female Chinese sturgeons, respectively over the study (Figure 3C). A similar decrease was also observed in the content of monocytes, from 17.2 to 15.0% in the male and 16.0 to 14.7% in the female in the same period of observations (Figure 3D). For the content of lymphocytes, the male Chinese sturgeon showed slight seasonal variation between 4.4 and 9.0%, whereas changes in the female were minimal between 6.5 and 8.1% (Figure 3E). There was also no statistical sex difference in differential leukocyte counts between both individuals.

The level of CRP in the blood of the two Chinese sturgeons showed different changes over the study. The female exhibited a decrease in CRP values from 22.5 to 24.1 mg/L in spring and summer to 7.0 and 8.9 mg/L in fall and winter, whereas the male registered lower CRP values between 1.1 and 8.7 mg/L in the first three seasons but increased and exceeded that of the female in winter (Figure 3F). A significantly lower CRP level was noted in the male than the female in summer, and the CRP level in fall was significantly lower than that in summer for both individuals (*p* < 0.05). However, a significant increase (*p* < 0.05) in CRP from fall to winter was registered in the male Chinese sturgeon.

### Changes in Liver Function Parameters

**FIGURE 4:**
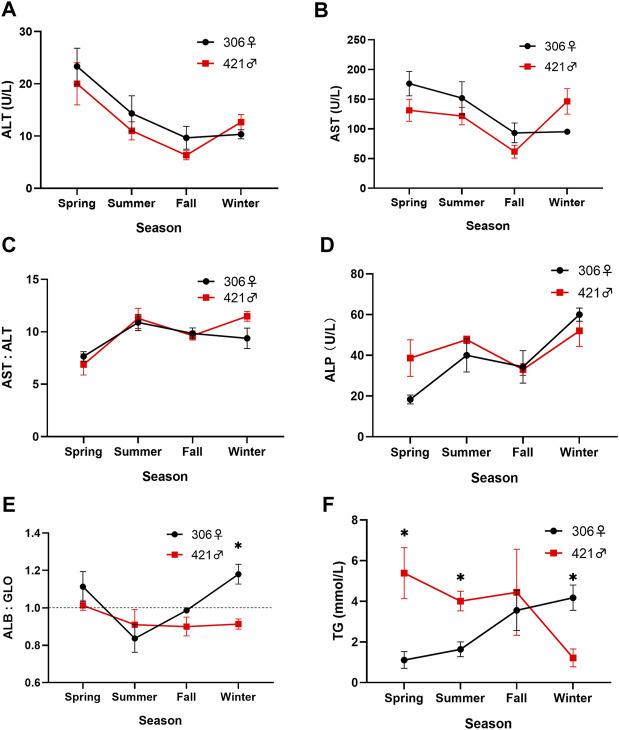
Seasonal variation of **(A)** alanine aminotransferase (ALT), **(B)** aspartate aminotransferase (AST), **(C)** transaminase ratio (AST:ALT), **(D)** alkaline phosphatase (ALP), **(E)** albumin to globulin ratio (ALB:GLO), and **(F)** triglycerides (TG) content in the blood of the two Chinese sturgeons. Data are mean ± SEM (*n* = 3), the seasonal data of sample size (*n* = 3) represents the three monthly data within one season. Significant difference (*p* < 0.05) in the parameter between the male and female individuals in the same season is denoted by an asterisk. Dashed line represents ratio of 1.0.

The level of alanine aminotransferase (ALT) in both Chinese sturgeons in different seasons was relatively similar and varied between 6.3 and 23.3 U/L (Figure 4A). The same was also noted in the level of aspartate aminotransferase (AST) for the two individuals, ranging between 61.7 and 176.0 U/L (Figure 4B). Both ALT and AST started a downward trend from spring and reached the lowest values in fall (Figures 4A,B). In particular, a significant decrease between spring and fall whereas a significant increase between fall and winter (*p* < 0.05) in the ALT and AST levels were noted in the male Chinese sturgeon. The transaminase (AST:ALT) ratio of both individuals was comparable and ranged between 6.9 and 11.5 (Figure 4C). A greater variation of this ratio was also apparent in winter, with a significant difference (*p* < 0.05) in values when comparing data among spring, summer, and winter.

The alkaline phosphatase (ALP) value of both individuals increased from 38.7 to 18.3 U/L in spring to 47.7 and 40 U/L in summer for the male and female Chinese sturgeons, respectively. Subsequently, the ALP level decreased to 33.0 and 34.3 U/L in fall, but peaked with an increase to 52 and 60 U/L for the male and female, respectively in winter (Figure 4D). The ALP value of the female was also significantly higher in winter than fall (*p* < 0.05); however, there was no sex difference in the activity of ALP between the two individuals in the same season.

The albumin (ALB) and globulin (GLO) values in the male Chinese sturgeon varied between 17.0 and 23.2 g/L, and 18.7 and 25.9 g/L, and in the female varied between 19.5 and 26.0 g/L, and 18.0 and 26.5 g/L, respectively over the study. Both individuals showed a decrease in the albumin to globulin ratio (ALB:GLO) from spring to summer, and the ratio of the female Chinese sturgeon was significantly lower at 0.84 in summer than 1.18 in winter (*p* < 0.05). During the study, the ALB:GLO value was lower in the male than the female except in summer, especially with significant lower level (*p* < 0.05) noted in winter (Figure 4E).

Seasonal changes in the level of triglycerides (TG) in the blood of both Chinese sturgeons showed an opposing trend, in which TG value decreased significantly from 5.39 mmol/L in spring to 1.22 mmol/L in winter for the male whereas TG value increased significantly from 1.11 mmol/L in spring to 4.18 mmol/L in winter for the female (*p* < 0.05). The TG level was also significantly higher in the male than the female in spring and summer. The reverse was noted in winter, with significantly higher TG level (*p* < 0.05) in the female than the male (Figure 4F).

## Discussion

### Seasonal Changes in Growth

The growth of fish differs from other animals which undergo distinct morphological stages in their life cycle, since their growth can be continuous until senescence as long as the food and environmental conditions are suitable (Xie, 2009). Under field conditions, Chinese sturgeon has a fast growth rate, with an average annual weight gain of 8.0–14.5 kg for females and 4.6–8.6 kg for males (Sichuan Changjiang Fisheries Research Institute, 1988). Similarly, under 21.0–22.5°C rearing conditions, the growth rate of body length and weight for postnatal female and male parents of Chinese sturgeon was 5.00 and 44.16%, and 3.23 and 23.30%, respectively over a study period of 9 months (Zhang et al., 2015). As suggested by the same authors, this faster growth rate may be related to the constant rearing temperature and the postnatal feeding of the parents to accumulate nutrients for the initiation of gonadal development. However, the average body length and weight of Chinese sturgeon at all ages do not increase regularly according to age, but rather decrease at some ages (Chen, 2007). Xie (2009) noted that under natural variable temperature conditions and due to the short suitable growth period throughout the year, the daily feeding rate of a 16 years old Chinese sturgeon in captivity ranged from 0.12 to 0.99%, with slow growth and loss in weight throughout the year, resulting in overall growth stagnation.

In this study, the change of body weight of the two captive Chinese sturgeons was different, in which the continuous growth of the female body weight can be due to the development of gonads, as suggested from the findings of Zhang *et al.* (2015). The increase in body weight and other growth-associated parameters in the female Chinese sturgeon can also be related to its relatively stable feeding rate over the rearing period except at the start of the study. In contrast, the male Chinese sturgeon tended to stop feeding in most summer and fall. Through analysis of gut content of recently deceased wild juvenile Chinese sturgeons from fisherman bycatch, Luo *et al.* (2008) revealed that less food items or empty stomachs were found in those samples mostly collected in the summer when the water temperature is higher. Similar findings of decrease or cessation in feeding were also observed in adult Chinese sturgeons reared in outdoor tanks during the warmer summer months (Wei et al., 2013). The decrease in body weight of the male Chinese sturgeon noted in the present study may thus be due to its higher sensitivity to the change in temperature, even though indoor water temperatures in summer can be a bit cooler relative to outdoor water temperatures, as noted in previous reports. The present findings also suggested that there could be sex difference in the response of change in water temperature in adult Chinese sturgeons. It was noted that the female Chinese sturgeon under study had significantly higher counter-flow swimming ratio than the male from spring to fall, implying that the female could be more active with stronger stamina than the male under warmer water temperature conditions.

### Innate Immune Parameters

Water temperature is a key factor affecting the solubility of oxygen in water, and oxygen is more soluble in cold water than in warm water; therefore, fewer erythrocytes are required to carry oxygen in fish in colder weather because oxygen is more readily available (Stolen et al., 1984). For example, Antarctic fish have fewer erythrocytes (Graham et al., 1985) and no changes in erythrocyte values were found in Atlantic salmon exposed to 5 and 15°C (Fivelstad et al., 2007). In agreement with previous reports, erythrocyte content of both Chinese sturgeons in this study also showed a decreasing trend after summer, when water temperature starts to fall.

Immune responses of poikilothermic animals, including fish, are known to vary seasonally, and in general, most immune parameters show increased levels in warmer summer time but tend to decrease towards colder winter months (Slater and Schreck, 1998; Bowden et al., 2007; (Slater and Schreck, 1998; Bowden et al., 2007; Papežíková et al., 2016). For example, as shown by Collazos *et al.* (1998) and De Pedro *et al.* (2005), seasonal changes in total leukocytes with significantly lower levels in spring and winter when compared with summer and fall were noted in male and female tench *Tinca tinca*. Morgan *et al.* (2008) also observed that the total leukocyte counts in the rainbow trout *O. mykiss* increased in spring, peaked in summer, and then decreased in fall to a minimum level in winter, coinciding with seasonal changes in water temperature and day length from spring to winter. In the common carp *Cyprinus carpio*, Engelsma *et al.* (2003) revealed that the relative number of circulating B lymphocytes in the total leukocyte population was significantly reduced when the fish was subjected to a single or multiple cold shocks. However, when inflammation occurred in fish, Lieschke and Trede (2009) noted an increase in level of neutrophils and leukocytes, indicating that lymphocytes are the key effector cells of adaptive immunity. An increase in total leukocytes is also associated with a decrease in blood flow to cardiac tissue (Afari and Bhat, 2016). Significantly higher level of leukocytes was also registered in fungal infected than healthy Caspian salmon *Salmo trutta caspius* from a fish farm study (Jamalzadeh et al., 2009). Although in the present study lymphocytes, neutrophils and monocytes showed either little seasonal variation or a slight decreasing trend from spring to winter in both Chinese sturgeons, their total leukocytes increased in fall and winter, suggesting that elevated leukocyte counts could be an indication of inflammation or infection in the two Chinese sturgeons during this study period.

C-reactive protein (CRP) is a homomeric membrane protein that can be irreversibly dissociated into five separate monomers at sites of inflammation and infection (Thompson et al., 1999). CRP is synthesized primarily by hepatocytes and to a lesser extent by other cells such as smooth muscle cells and macrophages, traditionally used as markers of infection and cardiovascular events. There is growing evidence that CRP serves an important function in the inflammatory process as well as in hose response to infection, including the complement pathway, apoptosis, phagocytosis and cytokine production (Sproston and Ashworth, 2018). Increases in CRP level have been noted in rainbow trout *O. mykiss* serum in response to exposure to anti-ectoparasitic chemicals used in aquaculture (Kodama et al., 2004). Sinha *et al.* (2001) also showed 3 to 5 fold increase in CRP concentrations in Indian major carp *Labeo rohita* under sublethal doses of chemical pollutants such as mercury and cadmium. In this study, it can be seen that CRP level in both Chinese sturgeons showed a significant decrease from summer to fall, indicating that CRP values can be greatly influenced by seasonality. However, the significantly higher CRP level of the female Chinese sturgeon than the male in summer and the increase in CRP values in the male from fall to winter suggested that the female could contract infection or inflammation in summer whereas similar problem may occur in the male in winter.

### Liver Function Parameters

Liver function in fish changes under stress conditions, and the levels of alanine aminotransferase (ALT) and aspartate aminotransferase (AST) activity are relevant stress indicators. Significant increases in these enzyme activities indicate enhanced transaminase processes and reflect stress-induced tissue damage in Indian major carp *L. rohita* (Philip et al., 1995). When zebrafish *Danio rerio* were exposed to sublethal concentrations of tributylin, Li and Li, 2021 also observed increasing levels of ALT and AST activities as well as ammonia and glucose in fish plasma, suggesting damage to fish hepatocytes and altered energy metabolism, which may, in turn, result in an increase in transaminase activity under toxic stress. In the present study, the levels of ALT and AST started a downward trend from spring and reached the lowest value in fall, and seasonal change did not negatively affect the liver function of both Chinese sturgeons based on the change of these two enzymes. The AST:ALT ratio is considered a non-invasive method for assessing liver fibrosis and cirrhosis, and a ratio of less than 1 indicates a good liver condition, while a ratio greater than 1 indicates a significantly damaged liver with compromised liver function (Giannini et al., 1999). During the present study, the transaminase ratios of both Chinese sturgeons in spring were significantly different from those in summer and winter, and the transaminase ratio was much greater than 1, with the maximum value reaching 11, indicating that the liver function of both Chinese sturgeons was significantly impaired. The degree of liver impairment also varied in different seasons, with the female showing the worst in summer whereas the male registering the highest ratio in winter.

Alkaline phosphatase (ALP) is a ubiquitous membrane-bound glycoprotein that catalyzes the hydrolysis of phosphate monoesters at alkaline pH. Alkaline phosphatase is classified into four isozymes based on the site of tissue expression, namely intestinal alkaline phosphatase, placental alkaline phosphatase, germ cell alkaline phosphatase and tissue non-specific alkaline phosphatase or liver/bone/kidney alkaline phosphatase (Sharma et al., 2014). In general, an elevated level of alkaline phosphatase means that the body’s liver function is impaired (Liu et al., 2018). In the present study, the ALP levels in both Chinese sturgeons were significantly higher in summer and winter, suggesting that their liver function may be negatively affected in some way when faced with high and low temperatures. Albumin is made in the liver and globulin by the body’s immune organs (Himmelfarb and McMonagle, 2001). When liver failure or cirrhosis occurs, the production of albumin as a molecule in the albumin to globulin ratio decreases, resulting in a low ALB:GLO value. Similarly, when antigens (enemies) such as hepatitis B virus exist in the body, the body’s immune organs have to increase their globulin level to defense against the enemies. In fish, increases in globulin level may also be due to toxic stress (Javed and Usmani, 2015). Thus, ALB:GLO ratio is an index used to track changes in serum or plasma composition and predict liver disorders, and its normal value lies between 1.1 and 2.5 in case of humans (Suh et al., 2014). Gopal *et al.* (1997) observed that when common carp *Cyprinus carpio* was treated with sublethal concentrations of heavy metals, its ALB:GLO ratio decreased significantly within the first 20 h of exposure. Javed *et al.* (2017) also found that under the exposure treatment to wastewater containing heavy metals, the serum ALB:GLO level of spotted snakehead *Channa punctatus* was lower in comparison to the control group. In this study, the ALB:GLO ratio in the male Chinese sturgeon was lower than 1.0 from summer to winter and similar low level was registered in the female in summer. The present findings thus imply that probable liver function impairment occurred in both individuals under the onset of higher temperature in summer; however, the symptom continued in the male even the temperature dropped in the fall and winter.

The triglyceride molecule is the major form of fatty acid storage and transport within the cells and in the plasma. The liver is the key organ for fatty acid metabolism. Fatty acids are biosynthesized and accumulated in the liver through the uptake of triglyceride molecules from the plasma by hepatocytes (Lebeck, 2014). However, low steady-state triglyceride concentrations in the liver can be attributed to a precise balance between the uptake of non-esterified fatty acids from plasma and *de novo* lipogenesis (Kawano and Cohen, 2013). Despite the high flux through these pathways, under normal conditions the liver stores only a small amount of fatty acids as triglycerides. In cases of over nutrition and obesity, hepatic fatty acid metabolism is altered, resulting in the accumulation of triglycerides in hepatocytes (Alves-Bezerra and Cohen, 2017). Latify *et al.* (2014) revealed that under heavy metal toxic stress, the Indian major carp *L. rohita* exhibited significant higher level of plasma triglycerides than the control group. Over the present study, although the triglyceride content of the male Chinese sturgeon was significantly higher than that of the female in spring and summer, the body weight of the male kept decreasing while that of the female showed a gradual increase. Such findings of an opposite trend of the change in the level of triglycerides in both individuals suggested that the liver fatty acid metabolism of the male Chinese sturgeon might have been disturbed, with impaired liver function and weight loss.

### Artificial Rearing of Chinese Sturgeons

As the population of wild Chinese sturgeons continues to decrease, captive breeding with subsequent release of the cultured juveniles into their natural habitat has become a main strategy and research focus in the conservation of Chinese sturgeons in the Yangtze River system. For instance, through telemetry tracking and recapture, Wang *et al.* (2014) revealed that the swimming migration ability for cultured sub-adult and juvenile Chinese sturgeons released in the Yangtze River was comparable to that of wild Chinese sturgeons, and in a post-release acoustic monitoring of hatchery-bred Chinese sturgeon juveniles, Wu *et al.* (2018) noted that the cultured juveniles adopted a similar distribution strategy to the wild Chinese sturgeon larvae in the Yangtze estuary area. However, Chinese sturgeons are highly sensitive and selective to the hydraulic environment of spawning grounds, and their spawning behavior is affected by various factors, including riverine hydraulic characteristics, water temperature, and the amount of sand in the river sediment (Wang and Xia, 2009). The setting of optimal environmental conditions for the artificial rearing of Chinese sturgeons has therefore become a critical issue in ongoing research. While evaluation studies on habitat suitability of Chinese sturgeons in the wild have been reported (Wang and Xia, 2008), presently there is still a lack of data on the best requirements for artificial culture setting in the laboratory.

Chinese sturgeons are benthic fish that prefer sandy bottom (Gu et al., 2008) and tend to spawn on gravel and pebble upstream river bed, with sediment diameter in the range of 50–500 mm (Yi et al., 2013). An increase in feeding frequency from 2 or 3 to 4 times a day has also resulted in faster growth in young Chinese sturgeons (Hu et al., 2020). In the present study, associated gravel and pebble were absent from the indoor rearing tank, which may not provide an optimal condition for the two Chinese sturgeons under study, therefore, the addition of gravel and pebbles obtained from rivers inhabited by Chinese sturgeon was considered for indoor culture. Similarly, provided that the amount of bait fed at each time is sufficient, whether increasing the daily feeding frequency from 3 to 4 times would enhance faster growth of adult Chinese sturgeons will remain to be investigated.

In the present study, the water quality in the indoor rearing tank fell within the suitable range for culture of Chinese sturgeons (Zhang et al., 2019), except for temperature. According to Zhu *et al.* (2006), the best growth performance of juvenile Chinese sturgeons under captivity was in water temperature between 18 and −25°C. The spring and winter water temperatures in the present indoor tank were around 15°C, which might therefore result in suboptimal condition for Chinese sturgeons under study. In particular, the male Chinese sturgeon appeared to be more sensitive to temperature change than the female as reflected by its negative specific growth rate over the study period. The present findings on hematological and serum biochemical parameters also revealed possible inflammation or infection as well as impaired liver function in both Chinese sturgeons. Hence, in future indoor culture, more attention should be paid to the changes of blood parameters in Chinese sturgeons, especially in the male individuals, daily monitoring of microorganisms and pathogenic bacteria in aquaculture water is also carried out. The immune and liver functions of wild Chinese sturgeons can be further tested in outdoor culture to verify whether the same problems exist and if seasonality/temperature or living environment can affect the health of Chinese sturgeons. The indoor rearing condition may also have potential impact on Chinese sturgeons and needs to be further optimized, such as increasing the size and depth of the culture tank, providing effective water temperature control, and formulating a nutritional diet for Chinese sturgeons. As well as trying to add flora and fauna from the wild environment to the indoor environment to increase the stability of the ecosystem. It is also suggested that routine veterinary care be provided so that immediate actions can be taken to safeguard the health of Chinese sturgeons during prolonged culture.

## Conclusion

Seasonal water temperature changes were found to adversely affect the growth of captive Chinese sturgeons, especially for the male. Seasonality also had an impact on innate immune parameters, including leukocytes and C-reactive protein, resulting in possible inflammation or infection. Through analysis of liver indicators, such as serum alanine aminotransferase, aspartate aminotransferase, transaminase ratio, alkaline phosphatase, albumin to globulin ratio and triglycerides, the liver function of Chinese sturgeons was impaired during winter. The male Chinese sturgeon under study appeared to be more susceptible to seasonal changes than the female. Albeit data were limited with two individuals, the present study called for the need to monitor changes of hematological parameters in Chinese sturgeons and provide routine veterinary care during prolonged culture.

## Data Availability

The original contributions presented in the study are included in the article/Supplementary Material, further inquiries can be directed to the corresponding authors.
